# Multiple Genotypes of the Commonly Co-Segregating Toll-Like Receptor 4 *Asp299Gly* and *Thr399Ile* in Baluchi Malaria Patients from Iran

**Published:** 2013-07-02

**Authors:** Sakineh Pirahmadi, Sedigheh Zakeri, Akram Abouie Mehrizi

**Affiliations:** Malaria and Vector Research Group (MVRG), Biotechnology Research Center (BRC ), Pasteur Institute of Iran, Tehran, Iran

**Keywords:** Malaria, Toll Like Receptor 4, Polymorphism, Iran

## Abstract

**Objective:**

Different studies have shown an association of *TLR4* polymorphisms with susceptibility/resistance to malaria disease. In the current immunogenetic study, we assessed the *TLR4* genotypes formed by the two common single nucleotide polymorphisms (SNPs) (*Asp299Gly* and *Thr399Ile*) in the co-segregate state in Baluchi *Plasmodium falciparum* infected and healthy populations from malaria hypoendemic areas of Iran. The study was performed to evaluate the distribution and correlation of *TLR4* co-segregating genotypes in patients with mild malaria. Moreover, the frequency of these genotypes was compared with reported results from other populations in similar or contrasting malaria settings around the world.

**Materials and Methods:**

In this case control study, the presence of 2 SNPs in the *TLR4* gene (*Asp299Gly* and *Thr399Ile*) were analyzed in 350 Baluchi patients with mild malaria and 350 unrelated healthy controls by using polymerase chain reaction/restriction fragment length polymorphism (PCR/RFLP) techniques followed by sequencing analysis. Differences in the *TLR4* co-segregate genotype frequencies among the studied group were determined by Fisher’s exact test.

**Results:**

Although the distribution of the two commonly co-segregating *TLR4* genotypes presented a diverse and distinct pattern in the Baluchi population, no significant difference was detected between the cases and controls (p>0.05). A lower frequency of *TLR4 Asp299Gly/Thr399Thr* was observed in Baluchis with mild malaria compared to African populations (p< 0.05).

**Conclusion:**

Differences in the co-segregation patterns of *TLR4 Asp299Gly/Thr399Ile* genotypes in the Baluchi population compared to other malaria endemic populations may suggest different local evolutionary pressure on *TLR4* polymorphisms by malaria in this region. The higher frequency of *Asp299Gly/Thr399Ile* genotypes among the Baluchi population compared with the African population (p< 0.05) which suffers from a larger number of severe cases might suggest that this genotype has a role in protecting against severe malaria. These findings are useful for further understanding the pathogenesis of severe malaria.

## Introduction

Malaria is one of the most important infectious diseases and every year 350-500 million cases of malaria are reported worldwide (1). The clinical manifestations of the disease are different among individuals in different malaria endemic settings of the world ranging from asymptomatic infection to severe life-threatening forms (2). This raises the question of why only a very small proportion of *Plasmodium*-infected individuals develop severe and complicated symptoms, while others remain asymptomatic or develop mild malaria.

Various studies have revealed the role of innate immune recognition in *Plasmodium* infection that releases inflammatory cytokines to clear the parasite from the circulation but may also contribute to disease severity (3-5). In addition, over the last decades, a variety of studies have focused on the discrepancy of different individuals in the protection or susceptibility to malaria and showed the interference of human genetic factors to this issue (6, 7). Cytokine expression also appears to be influenced by genetic factors and individuals may be classified as having a high or low inflammatory response (8, 9). More recently the role of different genes in susceptibility/resistance to severe malaria has been investigated. Among them the study of Toll-Like Receptors (TLRs) polymorphisms is currently attracting a great deal of attention (10).

TLRs are germline-encoded, transmembrane receptors, critical for the detection of bacteria, viruses, fungi and protozoa (3-5). *TLR4* is well known not only as a lipopolysaccharide (LPS) receptor (11, 12) but also as a receptor for other endogenous ligands and motifs from fungal, bacteria, mycobacteria and malaria parasites (13-16) that may activate the innate immune response. To date, more than 35 *TLR4* polymorphisms have been described (17) among which the most studied are two SNPs in the *TLR4* gene, *Asp299Gly* (rs4986790) and *Thr399Ile* (rs4986791) that are located in the leucine-rich repeat domain responsible for ligand recognition. These mutations affect the ligand-binding region (*Asp299Gly*) of *TLR4* and the co-receptor-binding region (*Thr399Ile*) of the receptor (18, 15). In addition, these *TLR4* polymorphisms have important functional consequences related to the production of pro- and antiinflammatory cytokines (19-21).

The prevalence of *TLR4* polymorphisms is different in various populations, possibly as a result of local infectious pressure and population migration. Sub-Saharan Africa has a high prevalence of the *Asp299Gly* polymorphism which possibly has protective effects against severe malaria (15). However, because of its effects in increasing susceptibility to severe bacterial infections, the *TLR4* haplotype containing only this SNP seems to have disappeared from Asian and American populations. In contrast, *Asp299Gly* has been found in co-segregation with *Thr399Ile* (15, 22). They have also been associated with infectious diseases, LPS hypo-responsiveness and cardiovascular disease (19, 20, 23).

Previous studies have assessed co-segregation of the *TLR4* genotypes in different populations around the world and reported variations in the prevalence of *TLR4* co-segregate genotypes (22, 24). This might reveal the effect evolutionary pressures in response to local infectious diseases and the subsequent susceptibility of a given population to those infections (22). For example, in African populations, where malaria exerts a strong evolutionary pressure, the *TLR4 Asp299Gly/Thr399Thr* genotype is more prevalent (22). In other studies protection against malaria mortality has been attributed to the *Asp299Gly/ Thr399Thr* genotype (15, 22). But in European populations, where the malaria pressure is lower than in the African population, the *TLR4 Asp299Gly/ Thr399Ile* genotype is more prevalent. Furthermore, in Asian and American populations these two SNPs are absent completely (22, 25, 26) as a result of specific evolutionary pressures that have depleted these polymorphisms in these populations (22). Therefore, it seems that the study of these two *TLR4* SNPs in the co-segregate state in different populations from various malaria endemic regions might aid understanding of the correlation of *TLR4* polymorphisms with clinical malaria as well as other infectious diseases.

In the present investigation, which is a continuation of our previous work (27), we analyzed and compared the prevalence of the *TLR4* genotypes formed by the two common SNPs (*Asp299Gly* and *Thr399Ile*) in the co-segregate state in mild malaria patients and healthy individuals in the Baluchi population from Iran where there has been no report of severe malaria cases. In addition, the frequency of the common *TLR4* co-segregate genotypes was compared between the Baluchi population who are living in malaria hypoendemic areas of Iran with those reported results from other populations from similar or contrasting malaria settings around the world. These results are used to obtain an insight into the evolutionary pressure of infections, particularly malaria on the *TLR4* polymorphisms in a population from the Middle East.

## Materials and Methods

### Study areas and population

The malaria endemic regions of Iran are situated in the south-eastern part of the country including the provinces of Sistan and Baluchistan, Hormozgan and Kerman. In Iran, the incidence of malaria has declined gradually over the last few years from 15,712 in 2007 to 3,015 cases in 2010 due to the beginning of elimination strategies (Center for Diseases Management and Control, Tehran, Iran, unpublished data). In these regions, there are no reports of anaemia, severe malaria or death due to malaria and most patients are adults with mild malaria. This study was carried out in the Chabahar District of the Sistan and Baluchistan Province in south-eastern Iran.

In this case-control study, the population-based controls were selected from healthy Baluchi individuals with no exposure or last exposure to malaria parasite during the past 10 years. Exposure status was obtained by interviewing the participant/or spouse or other family member, as well as searching the participant’s medical records in the malaria health center of the study area over the last 10 years.

Of the 700 Baluchi individuals who participated in this study, 350 with febrile *P. falciparum* were recruited from outpatient clinics at primary health centers in Chabahar district during 2003-2009. These patients, who presented with fever (in the preceding 48 hours with an axillary temperature ≥37.5˚C), or muscular pain and headache, were considered symptomatic and classified as having mild malaria. In addition, all patients had mono-infection with *P. falciparum*, previous history of malaria and parasitaemia ranging between 1,000 and 35,000 asexual parasites/mm3. The *P. falciparum* parasite species was diagnosed via microscopy locally in the study area and confirmed by nested PCR assay (28) in main laboratory, at the Pasteur Institute of Iran. Pregnant women and patients with severe medical disorders including diabetes mellitus, immunological disorders, and bacterial infections were excluded from the study. In addition, 350 healthy Baluchi individuals from the same study area with no history of febrile clinical symptoms of malaria at the time of blood sampling as well as no history of malaria during the last 10 years were included as controls. Nested PCR was also used to confirm the absence of infection in the control samples. In both infected and control groups, 1ml of blood was collected from the individuals in vacuum EDTA tubes and stored at -20˚C. Written informed consent was provided by all adult participants and by the parents or legal guardians of children and the study was approved by the Ethical Review Committee of Research in Pasteur Institute of Iran.

### Genotyping by PCR-RFLP

In this study two non-synonymous SNPs of *TLR4* (*Asp299Gly* and *Thr399Ile*) were typed in all 700 participants (case and control). These two SNPs were analyzed using PCR-RFLP analysis. Primers sequences and PCR conditions for these polymorphisms were reported in our previous study that assessed *TLR4* polymorphisms and also TLR9 and TIRAP polymorphisms in 640 Baluchi individuals (27). Briefly, 1 μl of template genomic DNA (10-50 ng) was amplified in a 25 μl volume containing 250 nM of primer concentration and a reaction mixture containing 50 mM KCl, 2 mM MgCl_2_, 10 mM Tris- HCl, 125 μM dNTPs (for each dNTP) and 0.2 U Taq polymerase (Invitrogen, Carlsbad, CA). *TLR4* Asp299Gly amplification yielded a 213 base-pair (bp) fragment. The PCR reaction consisted of one initial cycle at 95˚C for 5 minutes, 35 cycles at 94˚C for 1 minute, at 57˚C for 1 minute and 72˚C for 1 minute followed by 57˚C for 2 minutes and 72˚C for 10 minutes. For *TLR4 Thr399Ile*, amplification yielded a 185 bp fragment in which the PCR program consisted of one initial cycle at 95˚C for 5 minutes, 35 cycles at 94˚C for 1 minute, at 54˚C for 1 minute and 65˚C for 1 minute followed by 54˚C for 2 minutes and 65˚C for 10 minutes. The PCR products were electrophoresed on a 2% agarose gel (Invitrogen, Carlsbad, CA).

To screen the *TLR4 Asp299Gly* and *Thr399Ile SNPs*, the PCR products were cleaved using NcoІ and HinfІ (Fermentas) restriction endonucleases, respectively. The mixture was incubated at 37˚C overnight, and then electrophoresed on a 3% agarose gel (Invitrogen, Carlsbad, CA).

### DNA sequencing

Sequence analysis was carried out to evaluate the results obtained by RFLP, using the primers described previously (27). The PCR products were gel-puriﬁed using the Qiagen DNA puriﬁcation kit (Qiagen, Germany) according to the manufacturer’s instructions. DNA sequencing was done using the dideoxy chain termination procedure (Chemistry V3.1, Applied Biosystems) and the 3730XL DNA analyzer (Applied Biosystems) by MilliGen sequencing service (Labege, France).

### Statistical analysis

The sample size was calculated using the sample sized-unmatched case control by means of OpenEpi software (29). Differences in *TLR4* co-segregate genotype frequencies among the studied groups were determined by Fisher’s exact test using SPSS for windows (version 16.0). A p value of < 0.05 was considered to be significant. For comparison of the frequency of *TLR4* co-segregate genotypes that were observed in the Baluchi population with other populations from different parts of the world, the chi-square analysis was used.

## Results

**Fig 1:**
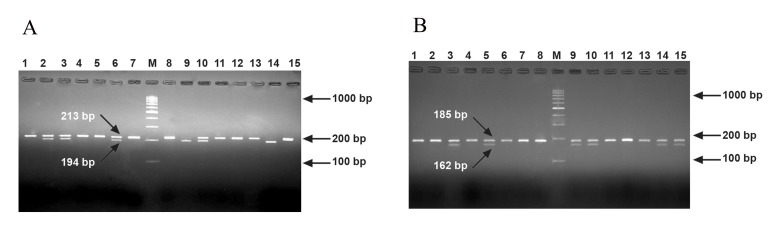
PCR-RPLF patterns of NcoI (A) and HinfI (B) digestion. The amplification products were digested by NcoI that digests the mutant allele of TLR4 Asp299Gly and HinfI that only digests the mutant allele of TLR4 Thr399Ile. The lane with the molecular weight marker (100 bp ladder) is labeled M. Lane 7 in (A) and 8 in (B) show uncut products as control.

Nested-PCR results showed that a total of 350 patients with mild malaria were infected with P. falciparum mono-infection and none of the unrelated healthy control participants had either *P. falciparum* or *P. vivax* infections. All of the studied participants were successfully assessed for *TLR4 Asp299Gly/Thr399Ile* SNPs (Fig 1) and sequencing data confirmed the RFLP results.

In the assessment of co-segregate *TLR4 Asp299Gly/ Thr399Ile* polymorphisms in infected individuals found the following distribution of genotypes; *Asp299Asp/Thr399Thr (80.6%), Asp299Gly/ Thr399Ile (9.4%), Asp299Asp/Thr399Ile (6%), Asp299Gly/Thr399Thr (2.6%)* and *Gly299Gly/ Thr399Thr (1.4%)*. In non-infected participants a similar distribution of genotypes; *Asp299Asp/ Thr399Thr (80.6%), Asp299Gly/Thr399Ile (6.6%), Asp299Asp/Thr399Ile (9.4%) and Asp299Gly/ Thr399Thr (3.4%)* was observed. *Gly299Gly/Ile399Ile, Asp299Asp/Ile399Ile, Asp299Gly/Ile399Ile* and *Gly299Gly/Thr399Ile* genotypes were not observed in the studied population.

In this investigation, the wild genotype of *TLR4 (Asp299Asp/Thr399Thr)* had the highest frequency in both studied groups and the mutant genotype *(Gly299Gly/Thr399Thr)* had the lowest frequency in only Baluchi infected subjects (Table1). The detected commonly co-segregation *TLR4* genotypes showed no significant difference among cases and controls in only Baluchi infected subjects (p>0.05, Table1).

### Comparison of TLR4 *Asp299Gly/Thr399Ile* cosegregate genotypes in the Baluchi population with that in populations from other malaria endemic areas

The result of this study showed that the Baluchi population had a diverse and distinct pattern of two commonly co-segregating *TLR4* genotypes.

The wild type of *TLR4 (Asp299Asp/ Thr399Thr)* was significantly lower in the Baluchi in comparison with the African and Asian populations (p< 0.05, Table 2). In addition, the *Asp299Gly/Thr399Ile* genotype was found significantly higher in the Baluchi population in comparison with the African populations from hyperendemic regions with severe malaria (p< 0.05, Table 2). Furthermore, the *Asp299Gly/ Thr399Thr* genotype was significantly lower in the Baluchi population in comparison with the Sudanese and Dogon populations (p< 0.05, Table 2).

**Table 1:** Genotype frequencies for commonly co-segregating TLR4 Asp299Gly/Thr399Ile in Baluchi P. falciparum infected and non-infected groups


TLR4 Asp299Gly/Thr399Ile	Infected (%) (n = 350)	Non-infected (%) (n=350)	P value*

**Wild/Wild**(Asp299Asp/Thr399Thr)	282 (80.6)	282 (80.6)	1.000
**Heterozygote/Heterozygote**(Asp299Gly/Thr399Ile)	33 (9.4)	23 (6.6)	0.210
**Wild/Heterozygote**(Asp299Asp/Thr399Ile)	21(6)	33 (9.4)	0.118
**Heterozygote/Wild**(Asp299Gly/Thr399Thr)	9 (2.6)	12 (3.4)	0.659
**Mutant/Wild**(Gly299Gly/Thr399Thr)	5 (1.4)	-	-


*; P value < 0.05 was considered to be significant.

**Table 2:** Comparison of the observed TLR4 co-segregate genotype frequencies in the Baluchi healthy population with other populations from malaria endemic regions


TLR4 co-segregate Genotypes in Baluchi healthy population (N=350)	Sudan* (N=101)	Cameroon* (N=142)	Tanzania* (N=121)	Dogon* (N=241)	*Fulani (N=243)	*Han Chinese (N=100)

Asp299Asp/Thr399Thr 80.6%****	90.1%	93.3%	92.6%	91.1%	97.5%	100%
	p=0.025	p=0.001	p=0.002	p< 0.0001	p< 0.0001	p< 0.0001
**Asp299Gly/Thr399Ile 6.6%**	0.5%	0.4%	1.7%	0.4%	-	-
	p=0.024	p=0.004	p=0.036	p< 0.0001		
**Asp299Asp/Thr399Ile 9.4%**	-	-	-	-	-	-
					
**Asp299Gly/Thr399Thr 3.4%**	9.4%	6.3%	5.8%	8.3%	2.5%	-
	p=0.030	p=0.216	p=0.285	p=0.015	p=0.629	


*; Results from Ferwerda et al. (22). The Chi-square test was used for comparing the genotype frequency in the Baluchi with other populations. P value < 0.05 was considered to be significant.

## Discussion

The clinical manifestation of malaria varies between individuals from diverse parts of the world (2) and only a small subset of Plasmodium- infected individuals develops life-threatening complications. To date, the exact mechanism underlying these differences has not been fully elucidated but different studies strongly suggest that the genetic make-up of the host plays a fundamental role (7) in addition to the environment and the parasite itself. The discovery of such a gene(s) might facilitate both a better understanding of the disease and the design of an efficient vaccine. Different studies have evaluated the association of *TLR4* polymorphisms with susceptibility/resistance to different diseases including malaria (15, 10). Indeed, it is very important to consider the *TLR4* genotypes formed by the *Asp299Gly* and *Thr399Ile* polymorphisms in the co-segregating state, because different genotype patterns have been observed in diverse human populations. These genotypes appear to change the receptor’s activity and alter susceptibility to infectious diseases including malaria (15, 30, 31). Therefore, in the current immunogenetic study, we assessed these two common *TLR4* polymorphisms in the co-segregate state in Baluchi *P. falciparum* infected and healthy populations from malaria hypoendemic areas of Iran to evaluate the distribution and correlation of *TLR4* co-segregating genotypes with mild malaria.

Several studies have revealed the specific geographical distribution of *TLR4* genotypes (22, 24, 31) that might be due to the pressure of different infectious diseases in that particular region. In the present study the co-segregation of the *TLR4* common SNPs (*Asp299Asp/Thr399Thr, Asp299Gly/Thr399Ile, Asp299Asp/ Thr399Ile, Asp299Gly/Thr399Thr and Gly299Gly/ Thr399Thr*) in Baluchi patients with mild malaria is reported for the first time. The findings are in agreement with a recent report by Loana et al. (24) in different Iranian ethnic groups. However, the distribution of the *TLR4* genotypes varies in different parts of the world. Asian populations lack all four *TLR4* genotypes, *Asp299Gly/Thr399Ile, Asp299Gly/ Thr399Thr, Asp299Asp/Thr399Ile and Gly299Gly/ Thr399Thr *(32). The *TLR4 Asp299Gly/Thr399Thr* genotype is more frequent in Africa, whereas the *TLR4 Asp299Gly/Thr399Ile*, which is completely absent in Asian populations (22, 25, 26), has been shown to have a higher frequency in Europe (22) and in our Baluchi population (6.6%).

Therefore, the presence of a greater number of multiple genotypes of the *TLR4* common SNPs in the Baluchi population in comparison to the African, and East Asian populations may suggest the presence of particular genotypes in this region that may indicate local evolutionary pressure on *TLR4* polymorphisms by infectious diseases including malaria.

In the assessment of the two *TLR4* polymorphisms in the co-segregate state, we found that the frequency of different *TLR4* co-segregating genotypes was not significantly different among Baluchi patients infected with mild falciparum malaria and Baluchi healthy individuals (p>0.05). This result suggests that different *TLR4 Asp299Gly/Thr399Ile* co-segregating genotypes might not be associated with mild malaria in our studied population.

The cytokine study of the profile in LPS-stimulated whole blood cultures by Ferwerda et al. (32) revealed that *TLR4 Asp299Gly/Thr399Thr* heterozygous cells released significantly higher TNF-α than the wild type *TLR4 Asp299Asp/Thr399Thr*. However, the double heterozygote genotype (*Asp299Gly/ Thr399Ile*) responded in a similar way to the wild type genotype (*Asp299Asp/Thr399Thr*). It has also been shown that the presence of the *TLR4 Gly299Gly/ Thr399Thr* genotype is associated with an increase in susceptibility to malaria but a reduction in mortality and cerebral malaria in the Ghanaian and Cameroonian populations (15, 22). In the present investigation, the low frequency of *TLR4 Gly299Gly/ Thr399Thr (1.4%)* among Baluchi infected individuals, its absence in healthy participants and the high frequency of *Asp299Asp/Thr399Thr* (80.6%) among the studied population from an area with no report of severe malaria or mortality due to malaria might suggest an association of these genotypes with clinical symptoms of mild malaria. This hypothesis needs to be supported by analyzing *TLR4* polymorphisms in more samples from diverse malaria hypoendemic regions.

The low frequency of the *TLR4 Asp299Gly/ Thr399Thr (3.4%)* genotype among the Baluchi population compared to some African populations (e.g. 9.4% in Sudanese inhabitants) may support a role for this genotype in susceptibility to severe malaria (15, 22). Mockenhaupt et al. (15) reported that the *TLR4 Asp299Gly* genotype was associated with an increased risk of severe malaria. Our findings might support their report, as the frequency of the *TLR4 Asp299Gly/Thr399Thr* genotype was observed to be significantly lower in Baluchi individuals with mild malaria. Nonetheless, this cosegregate genotype was significantly higher in the Sudanese and Dogon populations (p< 0.05) with a large number of severe malaria cases (22).

One of the most notable findings of this study was the higher frequency of the *Asp299Gly/ Thr399Ile* genotype (6.6%) among the Baluchi population than the African population (0.4-1.7%, p< 0.05). The significantly lower frequencies of this co-segregate genotype in the African population, which has a larger number of severe cases of malaria than the Baluchi population, suggests that the *Asp299Gly/Thr399Ile* genotype may help protect against severe malaria. However, functional studies are needed to draw final conclusions on the effect of the *Asp299Gly/Thr399Ile* genotype and its association with malaria severity in this population.

## Conclusion

In the present study, mixed patterns of *TLR4 Asp299Gly/ Thr399Ile* co-segregate genotypes were reported in the Baluchi population. Comparison with other malaria endemic populations suggests different local evolutionary pressures on *TLR4* polymorphisms by malaria or other infectious disease in this region. A significantly lower frequency of the *Asp299Gly/Thr399Thr* genotype in the Baluchi population in comparison with the Sudanese and Dogon populations (p< 0.05), which have a large number of severe malaria cases, might support a role for this genotype in susceptibility to severe malaria. These findings need to be extended to populations of different ethnicity from diverse malaria endemic regions for further understanding of the pathogenesis of this serious disease.
